# Mosquito Larvicidal Activity and Cytotoxicity of the Extracts of Aromatic Plants from Malaysia

**DOI:** 10.3390/insects14060512

**Published:** 2023-06-01

**Authors:** Huimei Lim, Sook Yee Lee, Lai Yee Ho, Nam Weng Sit

**Affiliations:** Department of Allied Health Sciences, Faculty of Science, Universiti Tunku Abdul Rahman, Bandar Barat, Kampar 31900, Malaysia; yzlim93@gmail.com (H.L.); sookyeelee08@hotmail.com (S.Y.L.); holy@utar.edu.my (L.Y.H.)

**Keywords:** *Aedes albopictus*, *Curcuma longa*, *Ocimum americanum*, *Petroselinum crispum*, Gas chromatography–mass spectrometry, lethal concentration, Vero cell

## Abstract

**Simple Summary:**

Mosquitoes are not only a nuisance, but most of them pose a threat to human beings as they can transmit pathogens that are harmful and may turn fatal, such as dengue, chikungunya, and Zika. This study was carried out to evaluate the larvicidal properties of the solvent extracts from three aromatic plants, *Curcuma longa* (turmeric), *Ocimum americanum* (hoary basil), and *Petroselinum crispum* (parsley)—against the mosquito vector of dengue, *Aedes albopictus*. Thereafter, the phytochemical profiles and the cellular toxicity of the extracts were evaluated. The results indicate that the hexane extracts of *O. americanum* and *P. crispum* had the greatest larvicidal activity. Among these two, *O. americanum* was shown to be less toxic to African monkey kidney cells and possessed an abundant amount of methyl eugenol, which is a phytochemical with known larvicidal activities. Overall, our findings indicate that these aromatic plants, especially *O. americanum*, may prove to be highly promising alternatives as bioinsecticides.

**Abstract:**

Despite ongoing control efforts, the mosquito population and diseases vectored by them continue to thrive worldwide, causing major health concerns. There has been growing interest in the use of botanicals as alternatives to insecticides due to their widespread insecticidal properties, biodegradability, and adaptability to ecological conditions. In this study, we investigated the larvicidal activity and cytotoxicity effects of solvent extracts from three aromatic plants—*Curcuma longa* (turmeric), *Ocimum americanum* (hoary basil), and *Petroselinum crispum* (parsley)—against *Aedes albopictus*. Subsequently, we examined the phytochemical composition of the extracts through gas chromatography–mass spectrometry (GC–MS) analysis. Results revealed that the hexane extracts of *O. americanum* and *P. crispum* exhibited the greatest larvicidal activity with the lowest median lethal concentration (LC_50_) values (<30 µg/mL) at 24 h post-treatment, with the former found to be significantly less toxic towards African monkey kidney (Vero) cells. The GC–MS analysis of the said extract indicated the presence of different classes of metabolites, including phenylpropanoids, very long-chain alkanes, fatty acids and their derivatives, and terpenes, with the most abundant component being methyl eugenol (55.28%), most of which, have been documented for their larvicidal activities. These findings provide valuable insights into the potential use and development of bioinsecticides, particularly from *O. americanum*.

## 1. Introduction

Dengue fever, Zika, and chikungunya are among the many mosquito-borne diseases that continue to pose significant threats to public health, particularly in tropical and subtropical regions. It was anticipated that close to half of the world’s population would be at risk of arboviral infections by the year 2050 [1]. Among all these, dengue stands out as the most rapidly spreading virus globally.

According to the World Health Organization, in the year 2019, the largest number of dengue cases were reported globally. All regions were affected, and dengue transmission was recorded in Afghanistan for the first time. The American region reported 3.1 million cases, with more than 25,000 cases classified as severe. High numbers of dengue cases were reported in Bangladesh (101,000), Malaysia (131,000), the Philippines (420,000), and Vietnam (320,000) in Asia [2,3]. Dengue infections have not only affected the well-being of the people, causing hospitalizations and deaths, but they have also led to the stagnation of the overall economy. As tropical regions are mostly comprised of developing countries, the impact of dengue outbreaks on the socio-economic sector of these regions may disrupt the already vulnerable societies, which will lead to them being less than able to cope with the added financial strain [4].

The primary vectors that transmit these arboviral diseases are the *Aedes* (*Stegomyia*) mosquitoes, primarily *Ae. aegypti* mosquitoes and, to a lesser extent, *Ae. albopictus*. In Malaysia, *Ae. aegypti* is the primary vector for dengue and Zika. On the other hand, *Ae. albopictus,* which serves as a secondary vector, plays an important role in the maintenance of dengue and chikungunya viruses in places where *Ae. aegypti* is absent or not well established. Their vectorial capacity is exemplified by their biting behavior as multiple feeders, in which they can bite several people consecutively when their feeding is disturbed [5]. Both of these *Aedes* species are prolific breeders in artificial as well as natural containers. As an adaptation to urban domestic habitats, the *Ae. aegypti* have begun exploiting a wider range of artificial containers such as vases, water tanks, and tires [6]. However, *Ae. albopictus* continues to be the most successful invasive species due to its greater ecological plasticity and stronger competitive aptitude. This mosquito species has already shown signs of adaptation to colder climates, which may result in disease transmission in new areas [7,8,9].

Vector-based interventions are the primary methods of control for most mosquito-borne diseases. Prior to the development of insecticides, these interventions mainly relied on environmental management, which included the removal of mosquito breeding sites and the installation of mosquito nets and screens in housing to prevent mosquito access through doors and windows. Eventually, the discovery and subsequent utilization of various synthetic insecticides took over as the main intervention strategy due to their rapid action and high efficacy. The active ingredients in synthetic chemicals such as organochlorine and organophosphate compounds are incorporated into bed nets, in indoor residual spraying, and also in outdoor space spraying and fogging [10,11].

However, numerous complications emerged due to the extended and prolonged usage of synthetic insecticides. Protopopoff et al. [12] reported that some mosquito species naturally avoid contact with insecticides, and there is evidence of the emergence and spread of resistance through metabolic detoxification. For instance, monooxygenases are often associated with metabolic resistance to pyrethroids, such as permethrin, while resistance to organophosphates and carbamates, such as bendiocarb, is incurred by the magnification of carboxyl-cholinesterase activity [13]. Despite sustained efforts to control mosquitoes, the spread of mosquito-borne diseases continues to threaten the health of billions of people worldwide and hamper economic development [14]. Moreover, chemical control using pyrethroids and organophosphates is often challenged by high costs, low community adoption, and slow operational implementation [15,16]. The continuous application of synthetic insecticides has also led to the biological magnification of toxic substances through the food chain due to their slow degradation, with adverse effects on environmental quality and non-target organisms, including humans [17].

As such, the application of eco-friendly alternatives through biological control methods has become the central focus of the control program in lieu of chemical insecticides. One of the most effective approaches under the biological control program is to explore floral biodiversity to source potential insecticides of botanical origin. Unlike conventional insecticides, which are based on a single active ingredient, plant-derived insecticides are mainly composed of botanical blends of various bioactive compounds. These may act concertedly on the vector’s behavioral and physiological processes, which will then leave very little room for the target vectors to develop resistance to such substances [18]. Moreover, botanical insecticides are preferred alternatives due to their rapid degradation and low toxicity [17]. The best approach to controlling mosquito populations is to target the aquatic larvae before to mature into terrestrial adults.

Turmeric (*Curcuma longa* L.) is a traditional Chinese herb belonging to the Zingiberaceae family [17]. It is a fragrant, perennial rhizomatous herb with large and pointed leaves [19]. The *C. longa* plant can grow up to 1 to 2 m in height with long, dotted leaves and funnel-shaped flowers. The yellowish egg- or pear-shaped rhizomes, which are grown underground, have a mild aroma with a sharp taste similar to ginger and are often used as a condiment and as coloring agents in medicines and food [20]. The constituents of the leaf, rhizome, and flowers of *C. longa* have been extensively studied [21,22,23]. The rhizome essential oil has been reported to exhibit antimicrobial, anti-cancer, insecticidal, larvicidal, repellent, and antioxidant activities [24,25,26,27,28,29].

Additionally, the leaf oil has also been reported to exhibit fumigant toxicity against beetles generally found in stored products [30]. The *C. longa* plant contains tetrahydrocurcumin, an odorless and heat-resistant antioxidant compound, and numerous polyphenolic compounds such as curcumin, eugenol, cinnamic acid, limonene, linalool, turmerone, and vanillic acid [31]. Available scientific evidence suggests that the essential oil and some constituents in the turmeric extracts were reportedly shown to possess potent larvicidal properties against *Aedes aegypti* [32], *Ae. albopictus* [33], *Anopheles gambiae* [19], *An. stephensi* [34], *Culex quinquefasciatus* [35], and *Cx. pipiens pallens* [17]. Nonetheless, the toxicities of these phytochemicals may vary depending on the origins of the plants and their target mosquito species [18,36].

*Ocimum americanum* L., also known as hoary basil, lime basil, or American basil, is a medicinal and aromatic annual herb in the family Lamiaceae. It has a strong citrus smell, and the plant can grow between 15 and 35 cm in height with elliptic, pointy leaves and white or pale lilac flowers [37]. Unlike *O. basilicum* (Thai basil), which is a popular aromatic herb incorporated in Thai and Vietnamese cuisines, *O. americanum* is not frequently used as a culinary herb but rather as herbal medicine [38]. The seeds are considered diuretics and can be used as a tonic. A decoction of the leaves of the plant can be taken for treating coughs, constipation, stomachaches, and dysentery, as well as a mouthwash for relieving toothache [38,39]. The plant has been shown to possess antimicrobial, antifungal, antiulcer, insecticidal, and larvicidal activities as well as wound-healing effects [40,41,42].

Having about 150 members, *Ocimum* is considered the largest genus in the Lamiaceae family and is known to possess a rich source of essential oils with various biological activities [43]. *O. americanum* exhibits varying phytochemical compositions depending on the solvent system used [38]. Inevitably, the choice of extraction method would highly depend on the chemical nature of the bioactive compounds of interest [41]. The major compounds found in the essential oil and various solvent extracts of *O. americanum* are said to be eugenol, methyl chavicol, 1,8-cineole, citral, linalool, camphor, monoterpenoids, flavonoids, polyphenols, and limonene [40,44,45,46]. The known insecticidal compounds found in the volatile oils of some *Ocimum* spp. are methyl cinnamate, methyl chavicol, and eugenol. Other chemicals that have been reported to have insect-repellent activity are thymol, carvacrol, camphor, caryophyllene oxide, cineole, limonene, and myrcene. However, the chemical components and essential oil levels of *Ocimum* may vary between species and cultivars and under different growing conditions [47,48].

Unlike the more pronounced members of the *Ocimum* genus, for instance, *O. basilicum*, *O. sanctum*, and *O. tenuiflorum*, which are immensely investigated for their mosquitocidal and repellency properties, there is minimal research and evidence to reflect on the larvicidal efficacy of the extracts and essential oils of *O. americanum* against mosquito vectors. Only recently, Narayanan et al. [49] reported on the bioactive components of the aqueous extracts of *O. americanum,* which showed significant larvicidal properties against *Ae. aegypti*, *An. stephensi*, and *Cx. quinquefasciatus*.

*Petroselinum crispum* (Mill.) Fuss is a bright green, biennial herb belonging to the family Apiaceae. It is commonly called parsley or garden parsley. The plant can grow up to 30 to 100 cm tall, with aromatic flat or curled leaves and small, yellow-green flowers [50]. Parsley was appreciated for its medicinal properties long before it was accepted as a vegetable, a garnish, and a seasoning and flavoring agent. It has been traditionally used for ailments and complaints of the gastrointestinal tract as well as the kidney and lower urinary tract [51]. Parsley seeds have been claimed to be antimicrobial, antiseptic, and antispasmodic [52]. The leaves possess anticoagulant activity and are used as an antitussive and as a treatment for dermatitis, poor vision performance, hemorrhoids, eczema, nosebleeds, amenorrhea, hypertension, and hyperuricemia [53,54,55,56].

The seeds of *P. crispum* produce high amounts of essential oils. According to the reports by Farzaei et al. [50] and Agyare et al. [57], the primary components and active compounds that are reportedly found in the essential oils and solvent extracts of parsley consist of myristicin, apiol, monoterpene hydrocarbons, eugenol, coumarins, phenolic compounds, and flavonoids, particularly apigenin, apiin, and 6”-acetylapiin. In another study, four compounds isolated and identified from the methanolic extract of *P. crispum*, which were pabulenol, oxypeucedanin, oxypeucedanin hydrate, and N-(2-phenylethyl) hexanamide, were shown to possess significant phototoxicity properties [58,59]. The essential oil of *P. crispum* has long been considered a potential bioinsecticide due to the many reported findings of its significant mosquitocidal, larvicidal, and repellency properties against mosquito vectors, primarily the dengue vector *Ae. aegypti* [60,61,62] and other less common species such as *Cx. pipiens, Culiseta longiareolata* [63], and *Ochlerotatus caspius* [64].

In Malaysia, these three aromatic herbs are commonly used not only in traditional medicine but also as culinary herbs. Despite the known larvicidal potential of these plants against many mosquito vectors, there is a lack of data on their mosquitocidal properties against another equally important dengue vector, *Ae. albopictus*. Additionally, plants are a rich, untapped pool of phytochemicals that may one day replace synthetic insecticides in mosquito control programs. Although numerous studies have documented the efficacy of plant extracts, most of these do not look into characterizing and determining the structures of the active compounds that are responsible for their larvicidal activity. Hence, there is a need for more isolation and purification work to discover the true potential of these phytochemicals as botanical larvicides. Moreover, it is essential to assess the safety of these plant extracts through cytotoxicity screenings before they are adopted in public health interventions.

## 2. Materials and Methods

### 2.1. Plant Materials

Three aromatic plants, *Curcuma longa* L., *Ocimum americanum* L., and *Petroselinum crispum* (Mill.) Fuss, were obtained from the wet markets in Kampar (Perak), Ipoh (Perak), and Cameron Highlands (Pahang), respectively. All plant species were identified by Dr. Hean Chooi Ong, a former professor and ethnobotanist affiliated with Universiti Malaya, Malaysia, and cross-checked using the World Flora Online database [65]. Specimen vouchers were prepared for *O. americanum* (UTAR/FSC/10/013) and *P. crispum* (UTAR/FSC/10/024) and were deposited at the Faculty of Science, Universiti Tunku Abdul Rahman (Kampar Campus), Malaysia.

### 2.2. Preparation of Plant Extracts

The fresh plant materials were cleaned thoroughly under running tap water to remove dirt and soil. The rhizomes of *C. longa* (250.89 g), the leaves of *O. americanum* (786.72 g), and the leaves and stems of *P. crispum* (603.09 g) were subjected to maceration sequentially using solvents of increasing polarity, i.e., hexane, chloroform (Qrec, Chonburi, Thailand), ethyl acetate, ethanol (Merck, Darmstadt, Germany), methanol (RCI Labscan, Bangkok, Thailand), and distilled water [66]. The macerations were performed at room temperature and with an agitation of 110 rpm for three cycles (one day/cycle). After filtration, the organic solvents were removed using a rotary evaporator, while the water extracts were lyophilized. All dry extracts were kept at −20 °C pending bioassay. The percentage of yield (*w*/*w*) was calculated using the following formula:

Percentage of yield = (Weight of dry extract)/(Weight of fresh plant material) × 100

### 2.3. Mosquito Sampling and Larvae Culturing

**Figure 1:**
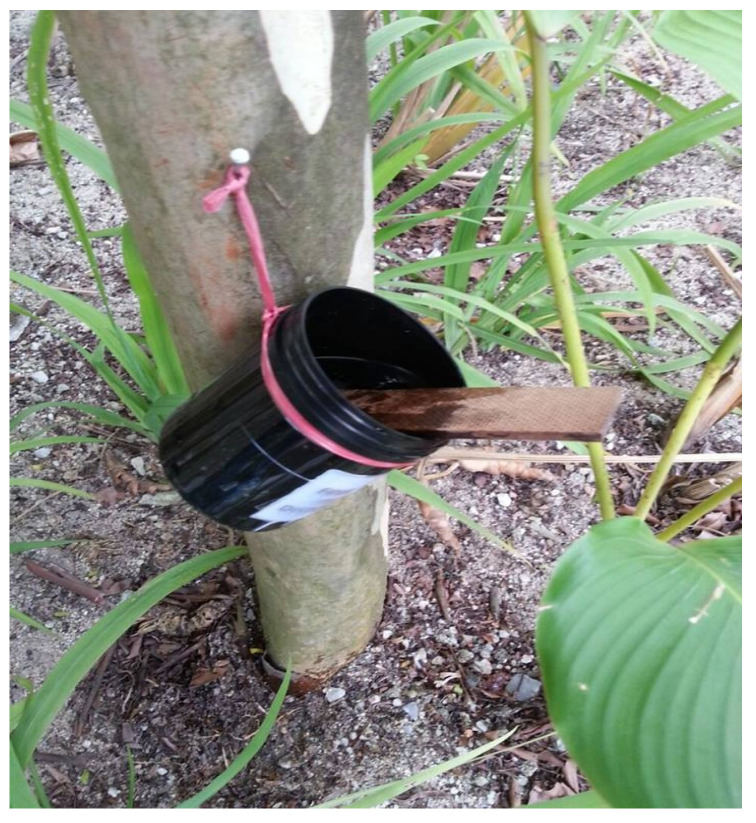
An ovitrap hung on a tree for mosquito oviposition.

Sixty ovitraps were set up inside the university campus (Kampar Campus) between October 2014 and January 2015 for mosquito egg collection. A hardboard paddle (10.0 cm × 2.5 cm × 0.3 cm) was inserted diagonally into each ovitrap (Figure 1). The ovitraps were hung on the trees approximately 1 m above the ground. The paddles were replaced, and the water was refilled three times a week. The egg-containing paddles were air-dried for two days before being immersed in plastic containers (18.0 cm × 17.0 cm × 7.0 cm) filled with dechlorinated tap water for egg hatching. The emerged larvae were fed with ground cat food (Cuties Catz, Perfect Companion (M) Sdn. Bhd., Kuala Lumpur, Malaysia). The third-instar larvae were then identified using a stereomicroscope based on their morphology [67]. Only larvae of the species *Ae. albopictus* were used for larvicidal bioassays.

### 2.4. Larvicidal Bioassay

The larvicidal bioassay was performed according to the World Health Organization (WHO) guidelines [68] with slight modifications. Each extract was dissolved in a dimethyl sulfoxide–ethanol mixture (3:2, *v*/*v*), sonicated for 1 min using an ultrasonic bath (S100H Elmasonic, Germany), and filtered using 0.45 µm nylon syringe filters to produce a stock solution of 60 mg/mL. Appropriate volumes of the stock solution were pipetted into round plastic containers (9.2 × 6.0 cm) filled with tap water to produce five different concentrations (50, 100, 200, 400, and 600 µg/mL) for the bioassay. The final volume for each container was 150 mL. Later, 20 third-instar larvae were introduced into each container, and larval mortality was observed and recorded at 2, 24, and 48 h post-treatment. The dimethyl sulfoxide–ethanol mixture in the containers was maintained at ≤1% to avoid any toxicity to the larvae. The larvae were considered dead if they did not move when prodded with a needle in the siphon or cervical region. A 1% dimethyl sulfoxide–ethanol mixture was used as a negative control, while temephos (Vector Control Research Unit, Universiti Sains Malaysia, Penang, Malaysia) at 1 µg/mL was used as a positive control. The bioassay was conducted in three replicates. The percentage of mortality was calculated using the following formula:

Percentage of mortality = (Number of dead larvae)/(Total number of larvae) × 100

### 2.5. Larvae Morphological Examination

The dead larvae treated with the hexane extracts of *O. americanum* and *P. crispum* at 600 µg/mL were examined for morphological abnormalities using a stereomicroscope.

### 2.6. Cytotoxic Activity

Healthy kidney epithelial cells derived from the African monkey (ATCC^®^CCL-81™) were used to assess the cytotoxicity of plant extracts. The cells were grown in supplemented Dulbecco’s Modified Eagle Medium (DMEM) at 37 °C and 5% carbon dioxide. The supplementation used was 10% fetal bovine serum, 10,000 U/mL of penicillin, 10 mg/mL of streptomycin, and 3.7 mg/mL of sodium carbonate [69]. Cell count was enumerated using a hemacytometer, and a cell concentration of 40,000 per well was seeded in a 96-well microplate. Subsequently, 100 µL of plant extract (5, 10, 20, 40, 80, 160, 320, and 640 µg/mL) was pipetted to the wells and incubated at 37 °C and 5% carbon dioxide for 72 h. Prior to this, the plant extract was diluted twofold serially in DMEM with 1% fetal bovine serum. Cell control and medium control were included in each microplate. After incubation, the viability of cells was ascertained using the protocol of the neutral red uptake assay, as described by Repetto et al. [70]. The absorbance value was measured at 540 nm using a microplate reader (Tecan, Switzerland) and used to determine the percentage of cell viability. A plot of the percentage of cell viability versus concentration was established for each extract, and the median cytotoxic concentration (CC_50_) was interpolated from the plot for the extract. The assessment was conducted in three replications.

### 2.7. Profiling of Metabolites Using Gas Chromatography–Mass Spectrometry (GC–MS)

The hexane extract of *O. americanum* was selected for metabolite profiling due to its potency for larvicidal activity. The qualitative composition analysis of the extract was performed using a gas chromatography–mass spectrometer (QP2010 Plus, Shimadzu Corporation, Kyoto, Japan). The separation of components was achieved using a fused silica capillary column (30.0 m × 0.25 mm × 0.25 µm) with 5% diphenyl-95% dimethylpolysiloxane (SLB-5ms, Supelco, Bellefonte, PA, USA). The column temperature was maintained at 50 °C for 3 min, ramped at a rate of 4 °C/minute to 300 °C, and held for 5 min. The total analysis time for each sample was 70.50 min. The helium gas (mobile phase) flowed at a linear velocity rate of 30 cm/minute. The extract was dissolved in acetone (HPLC grade, Fisher Scientific Limited, Loughborough, UK) at 2 mg/mL and filtered using a 0.22 µm nylon syringe filter prior to injection. The injector port temperature was set at 280 °C. The injection volume was 1 µL, and a split ratio of 10.0 was used for the sample injection. For the mass spectrometer settings, the ion source and interface temperatures were 200 °C and 300 °C, respectively. Electron impact ionization at 70 eV was used. The fragment ions of each component were monitored for an m/z of 40–600. The spectra of each component were compared with the spectra in the NIST 14 Mass Spectral Library and Search Software (National Institute of Standards & Technology, Gaithersburg, MD, USA), and the identity of the component with a matching quality >86% was reported. The analysis was performed in duplicate.

### 2.8. Data Analysis

The larval mortality rate was expressed as the mean ± standard deviation of three replicates. The data were subjected to Probit analysis to determine the median lethal concentration (LC_50_), 95% lethal concentration (LC_95_), upper confidence limit, lower confidence limit, and regression coefficient. The percentages of larval mortality and cell viability were tested for significance using one-way analysis of variance (ANOVA), followed by the post-hoc Duncan’s multiple range test. The statistical analysis was conducted using IBM SPSS Statistics for Windows, Version 23.0 (IBM Corp., Armonk, NY, USA). The significance level was set at α = 0.05.

## 3. Results

**Figure 2:**
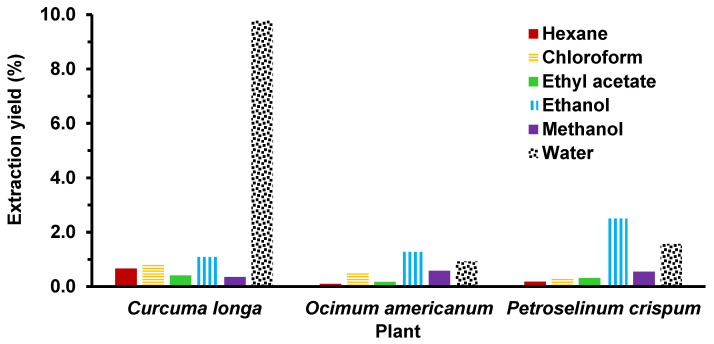
Yields of the extracts of aromatic plants obtained using sequential solvent extraction.

In this study, each aromatic plant sample was extracted sequentially using six solvents of increasing polarity, i.e., n-hexane (polarity index: 0.1), chloroform (4.1), ethyl acetate (4.4), ethanol (4.3), methanol (5.1), and water (10.2) [71]. This allows the segregation of plant secondary metabolites according to their polarity and facilitates the identification of an extract with specific biological activity. As shown in Figure 2, the yields for most of the extracts were less than 2% *w*/*w* except for the water extract of *C. longa* and the ethanol extract of *P. crispum,* which generated a yield of 9.77% and 2.50% *w*/*w*, respectively. The total yields for the extracts of *C. longa*, *O. americanum*, and *P. crispum* were 13.11%, 3.57%, and 5.40% *w*/*w*, respectively.

**Figure 3:**
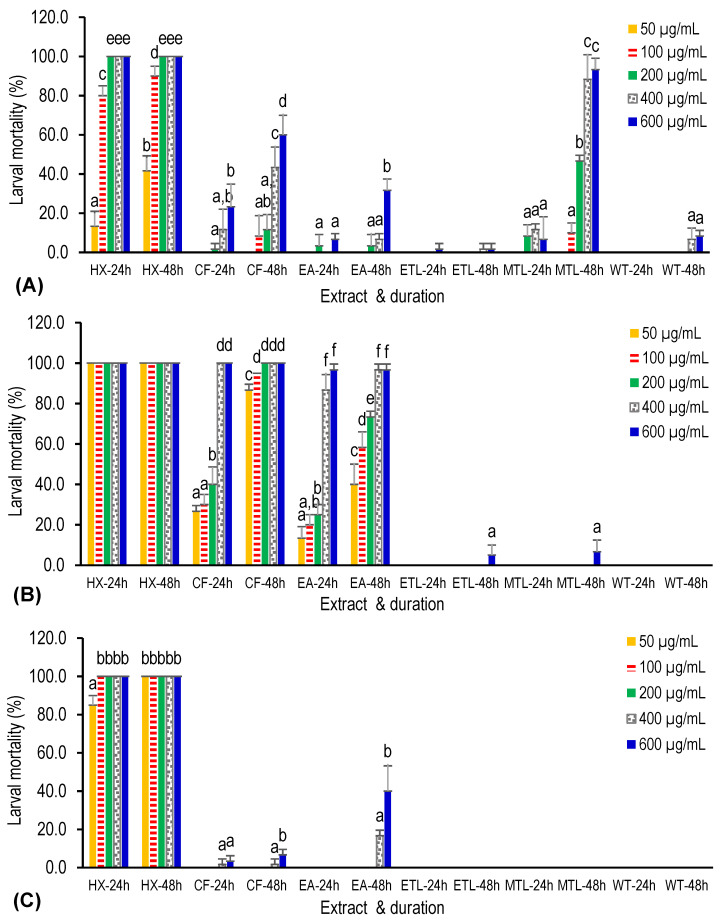
Percentages of larval mortality of *Aedes albopictus* treated with various extracts of aromatic plants, (**A**) *Curcuma longa*, (**B**) *Ocimum americanum*, and (**C**) *Petroselinum crispum,* for 24 and 48 h. The percentages are expressed as the means ± standard deviations of three replicates. Abbreviations: HX: hexane, CF: chloroform, EA: ethyl acetate, ETL: ethanol, MTL: methanol, and WT: water. Bars with the same alphabet (a–f) denote no significant difference (*p* > 0.05) using the one-way ANOVA test.

The 18 plant extracts were evaluated for larvicidal activity against third-instar larvae of *Ae. albopictus* at concentrations ranging from 50 to 600 µg/mL and the mortality rate was recorded at 2, 24, and 48 h post-treatment. The evaluation was performed in triplicate, with 20 larvae in each replicate. Since no mortality was observed in the negative control, Abbot’s correction was not applied in calculating the percentage of larval mortality. All extracts from the rhizomes of *C. longa*, except water extract, showed larvicidal activity at 24 h post-treatment. The hexane extract was the only extract that could kill all larvae within 24 h (100% mortality rate) when its concentration exceeded 200 µg/mL (Figure 3A). Although the water extract displayed larvicidal activity only at 48 h post-treatment, the mortality rate was less than 10%. Interestingly, the mortality rate for the methanol extract increased from 11.7 ± 2.9% to 88.3 ± 12.6% and from 6.7 ± 11.5% to 93.3 ± 5.8% for 400 µg/mL and 600 µg/mL, respectively, after an additional 24 h of treatment. These results suggest that the active larvicidal compounds may have a slow onset of action.

Among the three aromatic plants evaluated, *O. americanum* can be regarded as the plant with the strongest larvicidal activity, in terms of potency and onset of action, against the larvae of *Ae. albopictus*. In fact, the hexane extract of the leaves of *O. americanum* was the only extract found to have larvicidal activity within 2 h of treatment in the present study. It resulted in larval mortality rates of 10.0 ± 0.0% and 23.4 ± 7.6% at 400 and 600 µg/mL, respectively. The larvicidal effect of *O. americanum* was mainly documented from the hexane, chloroform, and ethyl acetate extracts, suggesting that the larvicidal compounds are non-polar or intermediate polar (Figure 3B). After 24 h of treatment, the hexane extract (≥50 µg/mL) produced a larval mortality rate of 100%. Consequently, further dilution beyond 50 µg/mL was performed for the hexane extract to obtain the LC_50_ and LC_95_ values. As shown in Table 1, the LC_50_ and LC_95_ values for the hexane extract at 24 h post-treatment were 26.60 µg/mL and 51.39 µg/mL, respectively.

As for *P. crispum*, only the hexane extract showed significant larvicidal activity. A larval mortality rate of 100% was observed at 24 h when the hexane extract concentration exceeded 50 µg/mL. The LC_50_ and LC_95_ values for this extract were 14.35 µg/mL and 83.66 µg/mL, respectively (Table 1). The three more polar extracts (ethanol, methanol, and water) were devoid of larvicidal activity (Figure 3C).

**Figure 4:**
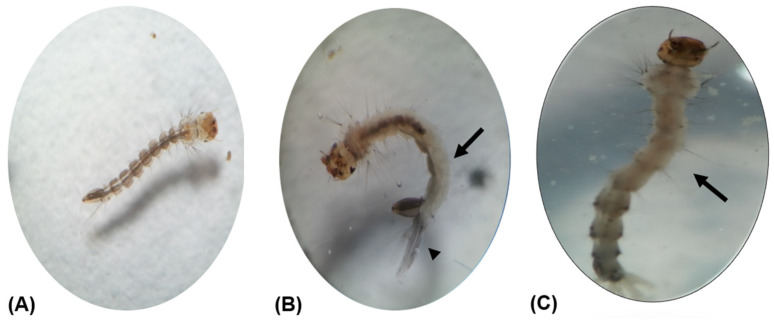
Morphological deformities of third-instar larvae of *Aedes albopictus* treated with aromatic plant extracts (40× magnification). (**A**) Normal, untreated larva; (**B**) Larva treated with the hexane extract of *Ocimum americanum* at 600 µg/mL; (**C**) Larva treated with the hexane extract of *Petroselinum crispum* at 600 µg/mL. Deformities such as a whitish abdomen (arrow) and a shrunken and pigmented cuticle of the anal papillae (arrowhead) were noticed in the larvae.

Unlike the normal untreated *Ae. albopictus* third-instar larva, which has a dark brown midgut and a pair of transparent sausage-like anal papillae (Figure 4A), larvae treated with the hexane extract of *O. americanum* had a whitish midgut with a shrunken and pigmented cuticle of anal papillae (Figure 4B). Similar abnormalities were also seen in the larvae treated with the hexane extract of *P. crispum* (Figure 4C).

**Figure 5:**
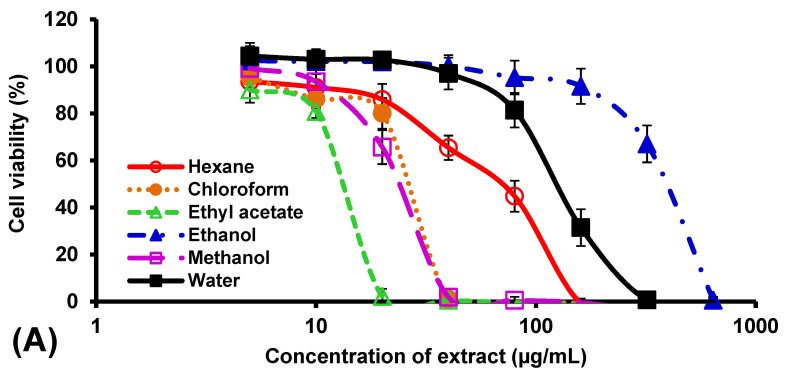

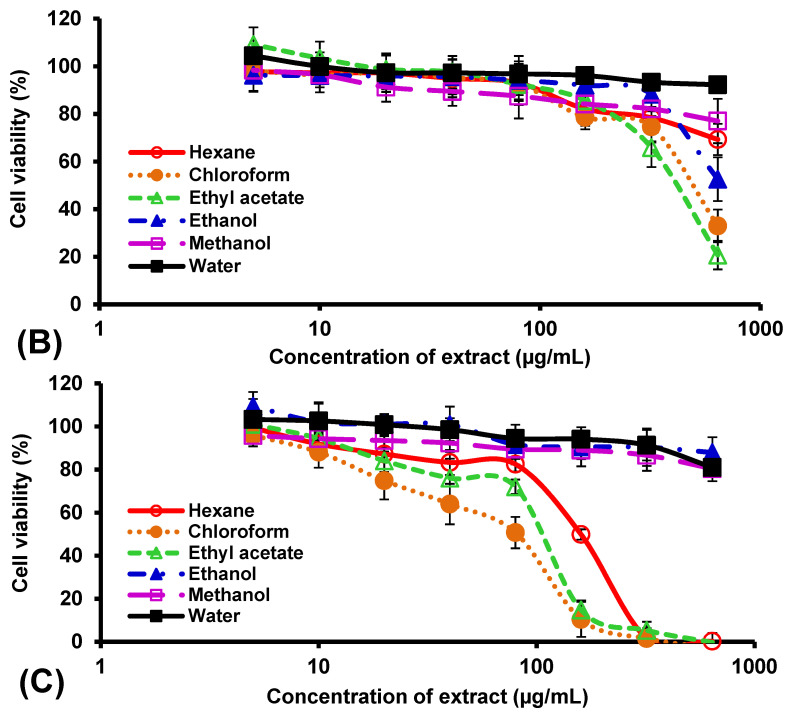
Viability of African monkey kidney epithelial (Vero) cells treated with various extracts from the aromatic plants (**A**) *Curcuma longa*, (**B**) *Ocimum americanum*, and (**C**) *Petroselinum crispum*. Each percentage is expressed as the mean ± standard deviation of three replicates. The *x*-axis is shown on the log scale.

Generally, the chloroform and ethyl acetate extracts of the three aromatic plants displayed higher toxicity towards Vero cells than the other four extracts, as shown in Figure 5. Only the water extract of *O. americanum* was not toxic to the Vero cells at all concentrations evaluated (*p* > 0.05). On the other hand, the ethyl acetate extract of *C. longa* exhibited the lowest CC_50_ (12.7 ± 0.6 µg/mL) among the extracts, indicating high toxicity toward the cells (Table 2). Notably, the methanol extract of *C. longa* also exerted strong toxicity (CC_50_ = 23.3 ± 1.5 µg/mL) towards the Vero cells, unlike the methanol extracts of the other two plants, which showed weak cytotoxicity with CC_50_ values higher than 640 µg/mL. This finding is also consistent with the significant larvicidal activity shown by the same extract at the 48-h exposure period (Figure 3A) compared to that of the other two plants.

**Figure 6:**
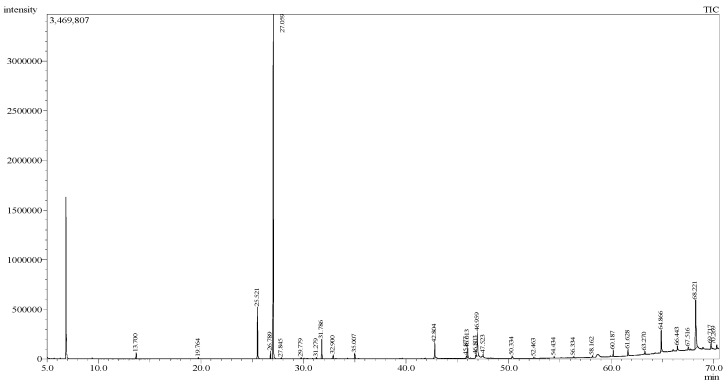
The total ion chromatogram for the hexane extract of *Ocimum americanum* leaves using gas chromatography–mass spectrometry.

For the two extracts with strong larvicidal activity in this study, the hexane extract of *O. americanum* showed much lower toxicity (CC_50_: >640 µg/mL) against Vero cells than the hexane extract of *P. crispum* (CC_50_: 159.3 µg/mL). Therefore, the hexane extract of *O. americanum* was selected for further analysis using gas chromatography–mass spectrometry to obtain more information on the potential larvicidal components present in the extract. The total ion chromatogram of the extract is shown in Figure 6. A total of 31 peaks were detected from the extract, and 14 of them were successfully identified, representing 92.07% of the total peak area or 91.95% of the total peak height (Table 3). The major component in the extract, based on the peak area, was methyl eugenol (55.28%), followed by tetrapentacontane (10.77%), eugenol (7.77%), linolenic acid (4.62%), and hexatriacontane (3.25%).

## 4. Discussion

Our findings on *C. longa* are consistent with the results reported in the literature. The essential oils of the rhizome of *C. longa* possess larvicidal activities against the disease-carrying mosquitoes *Ae. aegypti* [72,73], *An. gambiae* [19], *An. stephensi*, and *Cx. quinquefasciatus* [74]. The *C. longa* oil at 200 µg/mL could kill 84% of *Ae. albopictus* larvae within 24 h [32]. A larvicidal compound named ar-turmerone has been isolated from the ethyl acetate and ethanol extracts of the rhizome and is active against *Ae. aegypti* and *Cx. pipiens* [17,75].

To the best of our knowledge, this study is the first report of the larvicidal activity of *O. americanum* against *Ae. albopictus*. Several studies have evaluated the essential oils and solvent extracts derived from the leaves and/or stems of *O. americanum* for larvicidal activity against *Ae. aegypti* and reported LC_50_ values ranging from 15.03 µg/mL to 168 µg/mL for 24 h post-exposure [47,76,77,78]. Studies of the larvicidal activity of *P. crispum* have been largely focused on the essential oils of the seeds and fruits. The essential oils are active against the larvae of the mosquitoes *Ae. aegypti* [73], *Cx. pipiens*, *Cs. longiareolata* [63], and *Oc. caspius* [64]. Our results indicated that the hexane extract of the aerial parts of *P. crispum* could kill all *Ae. albopictus* larvae at 50 µg/mL after 48 h post-treatment. Based on the criterion proposed by Pavela [79], both hexane extracts of *O. americanum* and *P. crispum* have strong larvicidal activity, as their LC_50_ values for 24 h post-treatment were <50 µg/mL.

The morphological changes on the midgut and anal papillae of *Ae. albopictus* larvae in this study have also been observed when the larvae were exposed to the ethanol extract of papaya seed and the chloroform extract of the seaweed *Bryopsis pennata*, respectively [80,81]. In contrast, deformed larvae of *Ae. albopictus* with a blackish midgut and an extended cephalo–thoracic junction was found following treatment with the ethanolic leaf and fruit extracts of *Piper nigrum* [82] and the hexane leaf extract of *O. basilicum* [83]. These suggest that the midgut of mosquito larvae serves as a common target organ for the larvicidal effect of plant extracts, and the toxicity effects depend on the plant species, which is consistent with the findings reported by David et al. [84]. The morphological deformities are likely caused by the interference of hormonal control or interruption of chitin synthesis by the bioactive components in the extracts [85].

The cytotoxicity results of the present study were comparable to those of other published studies. Grover et al. [86] reported that the CC_50_ values of the ethanol and hydroalcoholic (methanol–water, 3:2 *v*/*v*) extracts for *C. longa* rhizomes were 525.0 and 16.8 µg/mL, respectively. Berrington and Lall [87] extracted the aerial parts of *P. crispum* using acetone solvent and obtained a CC_50_ value of 105 µg/mL against Vero cells. Although the cytotoxicity of the methanol extract of *O. americanum* using the same Vero cell line was similar, the CC_50_ values obtained for the chloroform (509.1 µg/mL), ethyl acetate (430.7 µg/mL), and ethanol (>640 µg/mL) extracts were much higher than those of our previous study, which recorded 86.3, 60.8, and 226.3 µg/mL, respectively [66]. This could be attributed to the source of origin of the *O. americanum* used in this study. The *O. americanum* in the present study was sourced from the Ipoh area, while the sample used in the previous study was obtained from the Kampar area. More studies are needed to confirm this observation.

Methyl eugenol and eugenol belong to the phenylpropanoids class of natural products, which are synthesized from primary metabolites, phenylalanine or tyrosine amino acids, via the shikimic acid pathway in plants [88]. They are common and major essential oil constituents of many *Ocimum* species, such as *O. basilicum*, *O. campechianum*, *O. gratissimum*, *O. sanctum*, and *O. urticifolium* [89,90]. Being the major component in the extract, methyl eugenol is likely to account for the potent larvicidal activity observed in this study. Methyl eugenol has been shown to have a biocidal effect against the mosquito larvae of *Ae. aegypti*, *Anopheles* spp., *Cx. pipiens pallens*, and *Ochlerotatus togoi* [91,92], albeit its activity against *Ae. albopictus* larvae is yet to be studied. On the other hand, eugenol has been reported to have larvicidal activity against *Ae. albopictus* with LC_50_ values of 28.1–67.4 µg/mL [93,94,95].

The gas chromatography–mass spectrometry analysis revealed that the leaves of *O. americanum* contained a substantial quantity of very long-chain hydrocarbons or alkanes, which are tetrapentacontane (C_54_H_110_) and hexatriacontane (C_36_H_74_), with abundances of 10.77% and 3.25%, respectively (Table 3). To the best of our knowledge, this is the first report of the presence of these alkanes in *Ocimum* species. Very long-chain alkanes are essential for the protection of plants from drought stress [96]. As the molecules only contain carbon and hydrogen atoms, they barely serve as bioactive metabolites, and hence, they may not account for the larvicidal activity of the extract.

The leaves of *O. americanum* also contained different types of fatty acids and their methyl or ethyl derivatives, which are linolenic acid (C18:3n3), palmitic acid (C16:0), linoleic acid (C18:2n6), methyl linolenate, and ethyl linolenate (Table 3). Similar fatty acids have been detected in the ethyl acetate or methanol extract of the leaves or aerial parts of *O. americanum* [41,97]. Among these components, the three fatty acids have been assessed against larvae of *Ae. albopictus,* and their LC_50_ values after 24 h of exposure were 71.34 µg/mL for linolenic acid, 40.96–85.61 µg/mL for palmitic acid, and 7.19–68.92 µg/mL for linoleic acid [98,99]. This indicates that the fatty acids could also contribute to the larvicidal property of *O. americanum* leaves.

Several terpenes were found in the hexane extract of *O. americanum* leaves as well, including elemol and β-elemene (sesquiterpenes), limonene (a monoterpene), and squalene (a triterpene), depending on the number of isoprene units present in the molecules. The two sesquiterpenes have been documented in the essential oils obtained from the leaves of *O. americanum* [100]. The β-elemene isolated from the essential oil of *Syzygium zeylanicum* is effective against *Ae. albopictus* larvae, with an LC_50_ value of 11.15 µg/mL after 24 h post-treatment [101]. In contrast, elemol was regarded as inactive against the larvae of *Ae. aegypti* and *Ae. albopictus* [102]. Despite being a minor compound with an abundance of <1%, limonene is known to have larvicidal activities against many mosquito species, i.e., *Ae. aegypti* [103], *Ae. albopictus*, *Cx. pipiens molestus* [104], *An. stephensi*, *Cx. quiquefasciatus* [105], *An. funestus*, and *An. arabiensis* [92]. Like limonene, squalene is only a minor compound in the hexane extract of *O. americanum* leaves. Nonetheless, it is not surprising to find the presence of squalene, as it is a metabolic intermediate of sterol biosynthesis in plants [106]. Squalene has been shown to have many biological properties, such as antioxidative, antibacterial, antifungal, anticancer, anti-inflammatory, cardioprotective, etc., as reviewed by Lozano-Grande et al. [106] and Lou-Bonafonte et al. [107]. However, further study is required to ascertain whether squalene possesses any mosquito larvicidal activity.

## 5. Conclusions

This present study showed that the rhizomes of *C. longa*, the leaves of *O. americanum*, and the leaves and stems of *P. crispum* possessed larvicidal activity against the third-instar larvae of *Ae. albopictus*. Among the aromatic plant extracts evaluated, the hexane extracts of *O. americanum* and *P. crispum* displayed the strongest larvicidal activity with the lowest LC_50_ values (<30 µg/mL) at 24 h post-treatment. Between these two hexane extracts, *O. americanum* was found to be significantly less toxic toward Vero cells than *P. crispum*. Gas chromatography–mass spectrometry analysis on the hexane extract of *O. americanum* indicated the presence of different classes of metabolites, including phenylpropanoids, very long-chain alkanes, fatty acids and their derivatives, and terpenes, with the most abundant component being methyl eugenol (55.28%). Some of the major components and minor components in the extract are known to be larvicidal compounds. Based on the findings from this study, further toxicity assessments of the potential active components, such as methyl eugenol, could be extended to aquatic organisms. This study also highlighted that aromatic plants are rich sources of botanical insecticides, which may play an important role in the ongoing efforts to reduce the mosquito population and the transmission of mosquito-borne diseases to humans. Hence, there is a possibility of exploring the potential of *O. americanum* leaves to be developed as a source of bioinsecticides.

## Figures and Tables

**Table 1:** Lethal concentrations and Probit analysis of larvicidal activities of aromatic plant extracts against third-instar larvae of *Aedes albopictus* after 24 and 48 h post-treatment.

Extract	Exposure Period (h)	LC_50_ (LCL-UCL) (µg/mL)	LC_95_ (LCL-UCL) (µg/mL)	Regression Coefficient (±Standard Error)	Chi-Square, X^2^	Degree of Freedom, df	*p*-Value
*Curcuma longa*							
Hexane	24	74.89 (68.16–82.21)	129.90 (113.39–159.84)	6.878 ± 0.872	0.120	3	0.989
	48	54.31 (46.24–61.19)	115.87 (97.29–157.62)	4.999 ± 0.820	0.185	3	0.980
Chloroform	48	483.23 (403.82–613.49)	2233.57 (1461.64–4464.62)	2.474 ± 0.319	3.796	3	0.284
Methanol	48	214.32 (189.82–240.98)	567.50 (475.36–720.79)	3.889 ± 0.360	1.879	3	0.598
*Ocimum americanum*							
Hexane	24	26.60 (20.63–35.53)	51.39 (37.72–117.40)	5.751 ± 0.656	15.568	5	0.008
	48	0.90	7.75	1.761 ± 1.475	0.753	5	0.980
Chloroform	24	143.04	650.19	2.501 ± 0.248	54.726	3	0.000
	48	18.77 (1.87–32.55)	85.30 (63.62–156.43)	2.502 ± 0.813	0.817	3	0.845
Ethyl acetate	24	198.42 (39.59–1338.13)	808.17 (334.96–2.46 × 10^9^)	2.697 ± 0.253	27.869	3	0.000
	48	74.78 (56.10–92.78)	501.23 (365.13–813.97)	1.991 ± 0.247	3.646	3	0.302
*Petroselinum crispum*							
Hexane	24	14.35 (0.54–28.62)	83.66 (57.86–163.92)	2.148 ± 0.736	3.110	3	0.375

LCL = Lower confidence limit; UCL = Upper confidence limit.

**Table 2:** Median cytotoxic concentrations of aromatic plant extracts were evaluated using African monkey kidney epithelial (Vero) cells.

Extract	Median Cytotoxic Concentration, CC_50_ (µg/mL)		
Plant	*Curcuma longa*	*Ocimum americanum*	*Petroselinum crispum*
Hexane	72.7 ± 6.7 ^b^	>640	159.3 ± 8.0 ^c^
Chloroform	26.7 ± 1.5 ^a^	509.1 ± 52.8 ^a^	82.7 ± 8.1 ^a^
Ethyl acetate	12.7 ± 0.6 ^a^	430.7 ± 40.1 ^a^	105.3 ± 4.5 ^b^
Ethanol	400.7 ± 33.5 ^d^	>640	>640
Methanol	23.3 ± 1.5 ^a^	>640	>640
Water	124.0 ± 9.5 ^c^	-	>640

The values are expressed as means ± standard deviations of triplicates. ‘-’ denotes no significant cytotoxicity even at the highest concentration tested (640 µg/mL). Mean values with different superscript letters (^a–d^) denote that the extracts are significantly different (*p* < 0.05) from each other by a one-way ANOVA or an independent-sample T-test.

**Table 3:** Metabolite profiling of the hexane extract of *Ocimum americanum* leaves using gas chromatography–mass spectrometry.

Peak	Retention Time (min)	Peak Area (%)	Peak Height (%)	Compound Name	Molecular Weight	Chemical Formula	Matching Quality (%)
1	13.700	0.79	1.00	_D_-Limonene	136	C_10_H_16_	93
2	19.764	0.18	0.22	Diketone alcohol	116	C_6_H_12_O_2_	89
3	25.521	7.77	8.43	Eugenol	164	C_10_H_12_O_2_	95
4	26.789	1.04	1.25	β-Elemene	204	C_15_H_24_	93
5	27.059	55.28	56.44	Methyl eugenol	178	C_11_H_14_O_2_	94
6	27.845	0.14	0.19	-	-	-	-
7	29.779	0.17	0.21	-	-	-	-
8	31.279	0.20	0.25	-	-	-	-
9	31.786	2.83	3.18	Elemol	222	C_15_H_26_O	93
10	32.900	0.59	0.65	-	-	-	-
11	35.007	1.07	0.90	-	-	-	-
12	42.804	2.61	2.43	Palmitic acid	256	C_16_H_32_O_2_	94
13	45.877	0.40	0.40	-	-	-	-
14	46.013	1.32	1.58	Methyl linolenate	292	C_19_H_32_O_2_	93
15	46.803	0.42	0.40	cis, cis-Linoleic acid	280	C_18_H_32_O_2_	88
16	46.959	4.62	3.99	Linolenic acid	278	C_18_H_30_O_2_	95
17	47.523	0.29	0.45	Ethyl linolenate	306	C_20_H_34_O_2_	86
18	50.334	0.25	0.36	-	-	-	-
19	52.463	0.14	0.20	-	-	-	-
20	54.434	0.21	0.26	-	-	-	-
21	56.334	0.15	0.21	-	-	-	-
22	58.162	0.23	0.28	-	-	-	-
23	60.187	0.90	1.08	Squalene	410	C_30_H_50_	89
24	61.628	0.89	1.03	-	-	-	-
25	63.270	0.40	0.48	-	-	-	-
26	64.866	3.25	3.49	Hexatriacontane	450	C_36_H_74_	94
27	66.443	0.62	0.68	-	-	-	-
28	67.516	0.46	0.47	-	-	-	-
29	68.221	10.77	8.01	Tetrapentacontane	758	C_54_H_110_	94
30	69.717	1.10	0.82	-	-	-	-
31	70.269	0.91	0.66	-	-	-	-

‘-’ denotes unable to be identified due to the matching quality <86%.

## Data Availability

All data are presented within the article.
